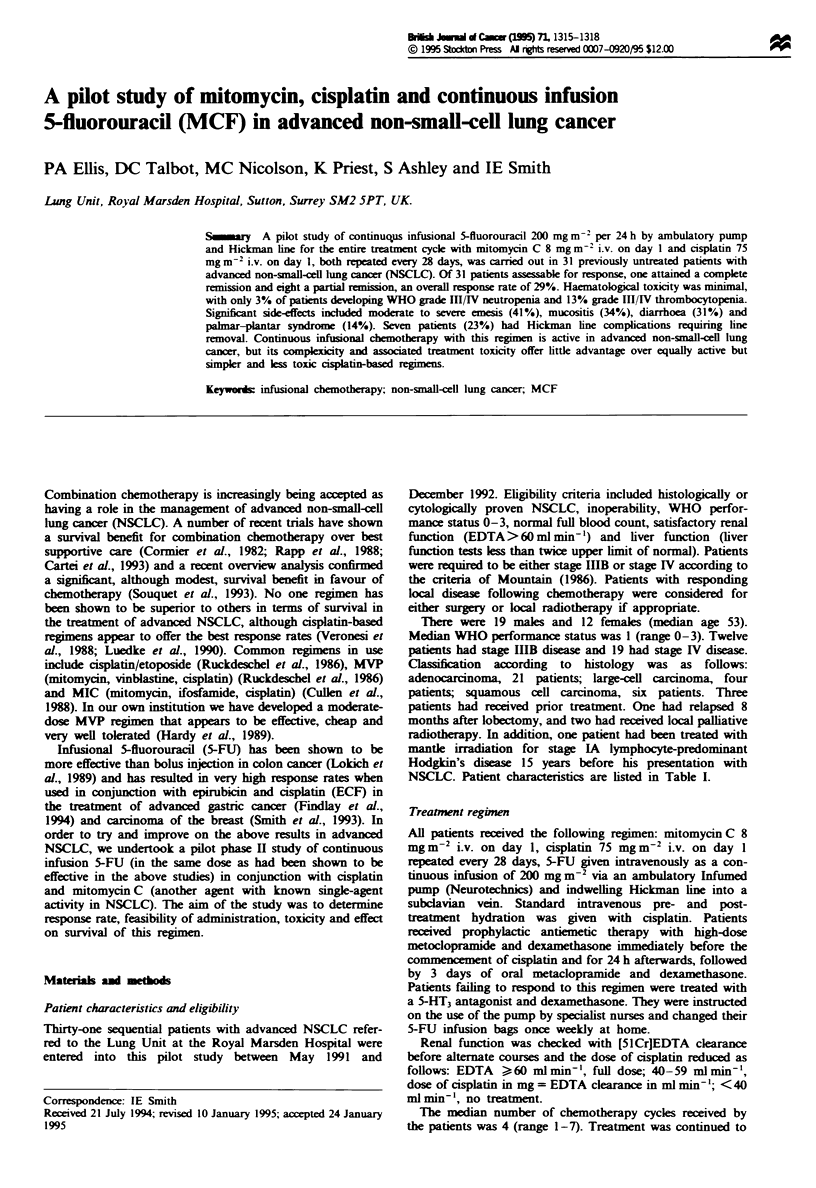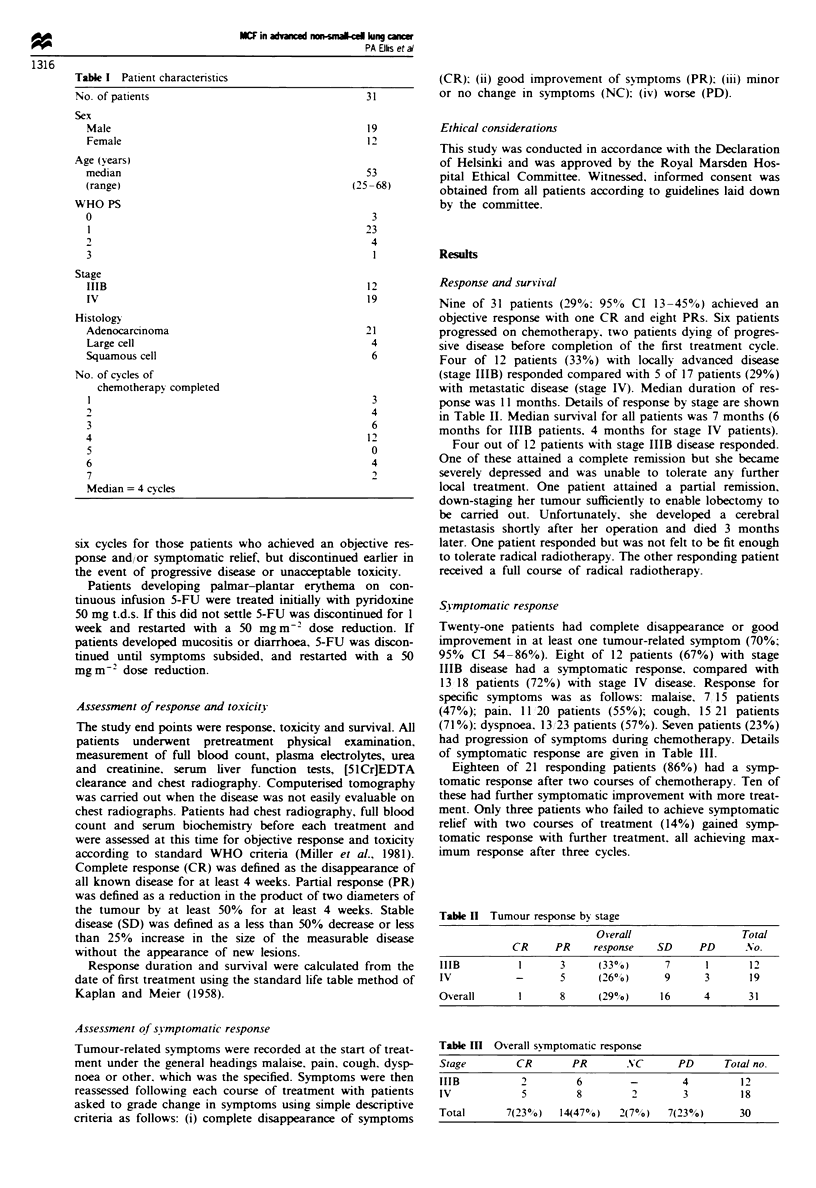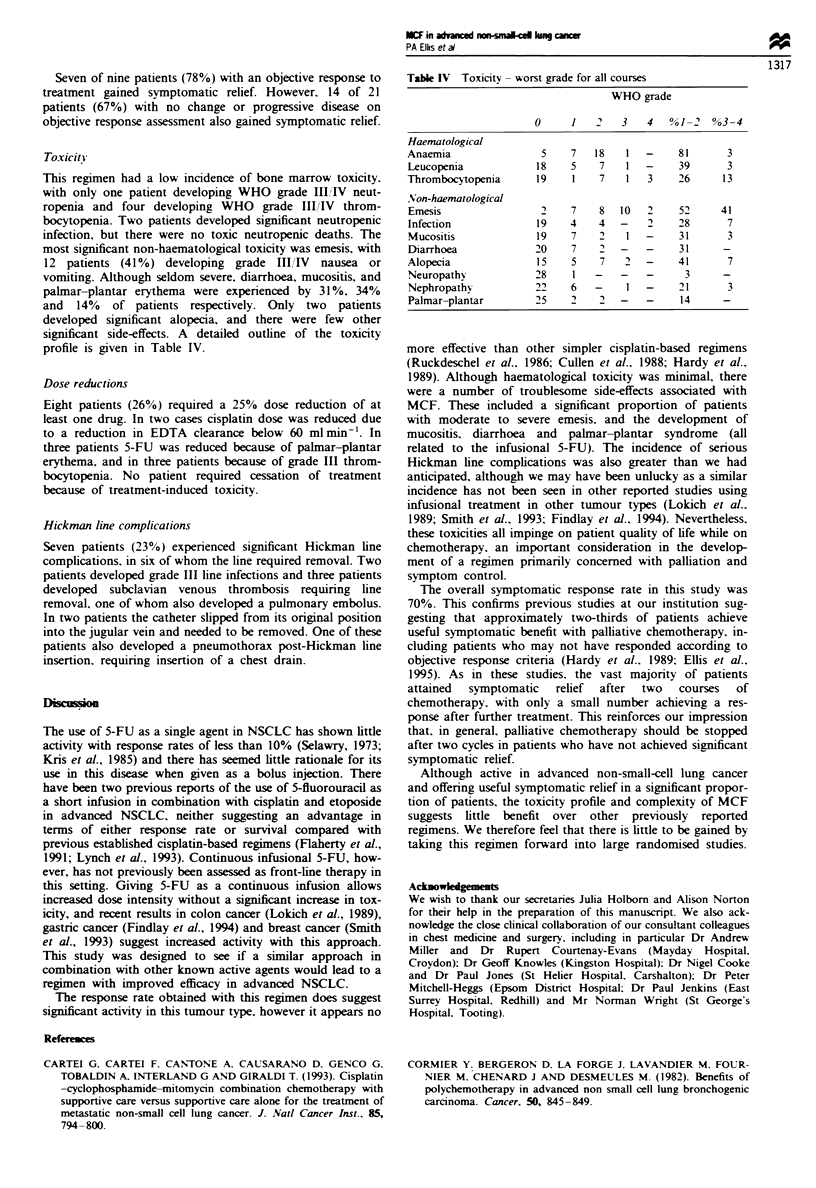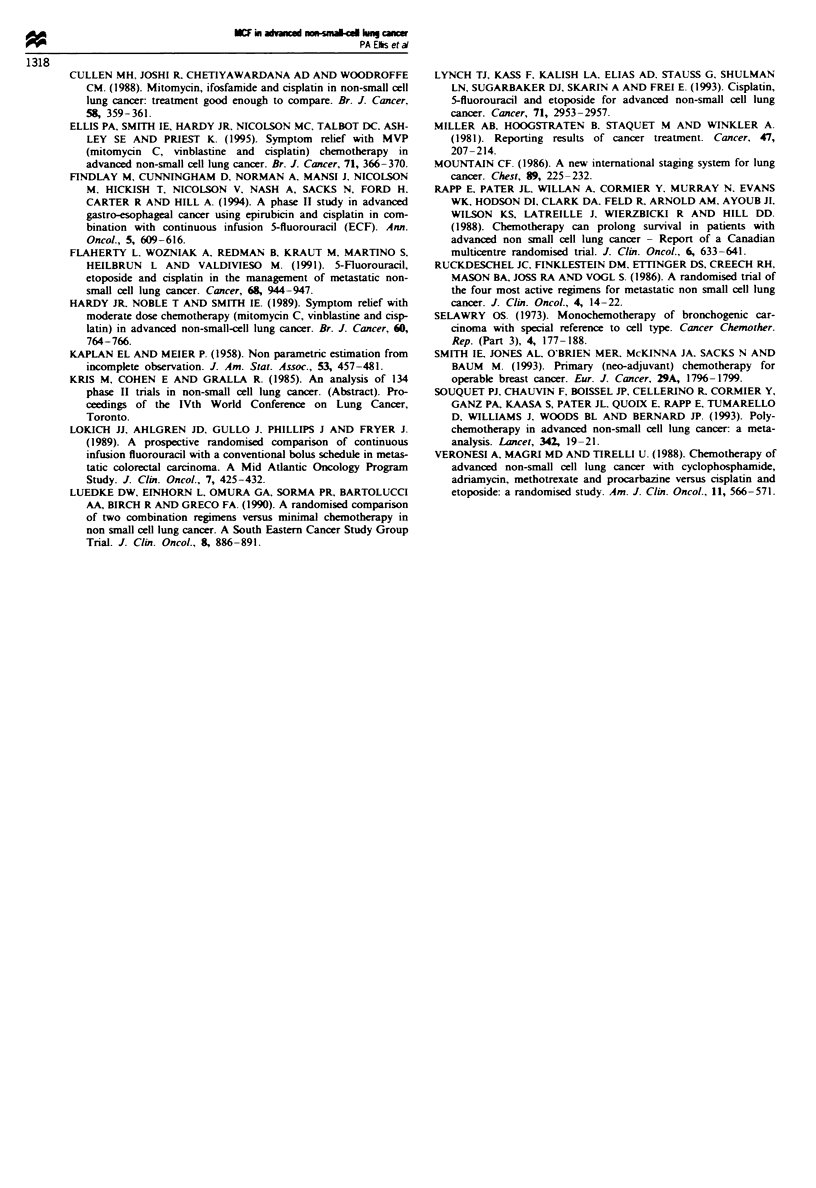# A pilot study of mitomycin, cisplatin and continuous infusion 5-fluorouracil (MCF) in advanced non-small-cell lung cancer.

**DOI:** 10.1038/bjc.1995.255

**Published:** 1995-06

**Authors:** P. A. Ellis, D. C. Talbot, M. C. Nicolson, K. Priest, S. Ashley, I. E. Smith

**Affiliations:** Lung Unit, Royal Marsden Hospital, Sutton, Surrey, UK.

## Abstract

A pilot study of continuous infusional 5-fluorouracil 200 mg m-2 per 24 h by ambulatory pump and Hickman line for the entire treatment cycle with mitomycin C 8 mg m-2 i.v. on day 1 and cisplatin 75 mg m-2 i.v. on day 1, both repeated every 28 days, was carried out in 31 previously untreated patients with advanced non-small-cell lung cancer (NSCLC). Of 31 patients assessable for response, one attained a complete remission and eight a partial remission, an overall response rate of 29%. Haematological toxicity was minimal, with only 3% of patients developing WHO grade III/IV neutropenia and 13% grade III/IV thrombocytopenia. Significant side-effects included moderate to severe emesis (41%), mucositis (34%), diarrhoea (31%) and palmar-plantar syndrome (14%). Seven patients (23%) had Hickman line complications requiring line removal. Continuous infusional chemotherapy with this regimen is active in advanced non-small-cell lung cancer, but its complexity and associated treatment toxicity offer little advantage over equally active but simpler and less toxic cisplatin-based regimens.


British Journal of Cancer (1995) 71, 1315-1318

© 1995 Stockton Press All rights reserved 0007-0920/95 $12.00

### A pilot study of mitomycin, cisplatin and continuous infusion 5-fluorouracil (MCF) in advanced non-small-cell lung cancer

PA Ellis, DC Talbot, MC Nicolson, K Priest, S Ashley and IE Smith

*Lung Unit, Royal Marsden Hospital, Sutton, Surrey SM2 5PT, UK.*

**Summary** A pilot study of continuous infusional 5-fluorouracil 200 mg m$^{-2}$ per 24 h by ambulatory pump and Hickman line for the entire treatment cycle with mitomycin C 8 mg m$^{-2}$ i.v. on day 1 and cisplatin 75 mg m$^{-2}$ i.v. on day 1, both repeated every 28 days, was carried out in 31 previously untreated patients with advanced non-small-cell lung cancer (NSCLC). Of 31 patients assessable for response, one attained a complete remission and eight a partial remission, an overall response rate of 29%. Haematological toxicity was minimal, with only 3% of patients developing WHO grade III/IV neutropenia and 13% grade III/IV thrombocytopenia. Significant side-effects included moderate to severe emesis (41%), mucositis (34%), diarrhoea (31%) and palmar–plantar syndrome (14%). Seven patients (23%) had Hickman line complications requiring line removal. Continuous infusional chemotherapy with this regimen is active in advanced non-small-cell lung cancer, but its complexicity and associated treatment toxicity offer little advantage over equally active but simpler and less toxic cisplatin-based regimens.

**Keywords:** infusional chemotherapy; non-small-cell lung cancer; MCF

Combination chemotherapy is increasingly being accepted as having a role in the management of advanced non-small-cell lung cancer (NSCLC). A number of recent trials have shown a survival benefit for combination chemotherapy over best supportive care (Cormier *et al.*, 1982; Rapp *et al.*, 1988; Cartei *et al.*, 1993) and a recent overview analysis confirmed a significant, although modest, survival benefit in favour of chemotherapy (Souquet *et al.*, 1993). No one regimen has been shown to be superior to others in terms of survival in the treatment of advanced NSCLC, although cisplatin-based regimens appear to offer the best response rates (Veronesi *et al.*, 1988; Luedke *et al.*, 1990). Common regimens in use include cisplatin/etoposide (Ruckdeschel *et al.*, 1986), MVP (mitomycin, vinblastine, cisplatin) (Ruckdeschel *et al.*, 1986) and MIC (mitomycin, ifosfamide, cisplatin) (Cullen *et al.*, 1988). In our own institution we have developed a moderate-dose MVP regimen that appears to be effective, cheap and very well tolerated (Hardy *et al.*, 1989).

Infusional 5-fluorouracil (5-FU) has been shown to be more effective than bolus injection in colon cancer (Lokich *et al.*, 1989) and has resulted in very high response rates when used in conjunction with epirubicin and cisplatin (ECF) in the treatment of advanced gastric cancer (Findlay *et al.*, 1994) and carcinoma of the breast (Smith *et al.*, 1993). In order to try and improve on the above results in advanced NSCLC, we undertook a pilot phase II study of continuous infusion 5-FU (in the same dose as had been shown to be effective in the above studies) in conjunction with cisplatin and mitomycin C (another agent with known single-agent activity in NSCLC). The aim of the study was to determine response rate, feasibility of administration, toxicity and effect on survival of this regimen.

### Materials and methods

### Patient characteristics and eligibility

Thirty-one sequential patients with advanced NSCLC referred to the Lung Unit at the Royal Marsden Hospital were entered into this pilot study between May 1991 and December 1992. Eligibility criteria included histologically or cytologically proven NSCLC, inoperability, WHO performance status 0–3, normal full blood count, satisfactory renal function (EDTA > 60 ml min$^{-1}$) and liver function (liver function tests less than twice upper limit of normal). Patients were required to be either stage IIIB or stage IV according to the criteria of Mountain (1986). Patients with responding local disease following chemotherapy were considered for either surgery or local radiotherapy if appropriate.

There were 19 males and 12 females (median age 53). Median WHO performance status was 1 (range 0–3). Twelve patients had stage IIIB disease and 19 had stage IV disease. Classification according to histology was as follows: adenocarcinoma, 21 patients; large-cell carcinoma, four patients; squamous cell carcinoma, six patients. Three patients had received prior treatment. One had relapsed 8 months after lobectomy, and two had received local palliative radiotherapy. In addition, one patient had been treated with mantle irradiation for stage IA lymphocyte-predominant Hodgkin's disease 15 years before his presentation with NSCLC. Patient characteristics are listed in Table I.

### Treatment regimen

All patients received the following regimen: mitomycin C 8 mg m$^{-2}$ i.v. on day 1, cisplatin 75 mg m$^{-2}$ i.v. on day 1 repeated every 28 days, 5-FU given intravenously as a continuous infusion of 200 mg m$^{-2}$ via an ambulatory Infumed pump (Neurotechnics) and indwelling Hickman line into a subclavian vein. Standard intravenous pre- and post-treatment hydration was given with cisplatin. Patients received prophylactic antiemetic therapy with high-dose metoclopramide and dexamethasone immediately before the commencement of cisplatin and for 24 h afterwards, followed by 3 days of oral metaclopramide and dexamethasone. Patients failing to respond to this regimen were treated with a 5-$HT_3$ antagonist and dexamethasone. They were instructed on the use of the pump by specialist nurses and changed their 5-FU infusion bags once weekly at home.

Renal function was checked with [51Cr]EDTA clearance before alternate courses and the dose of cisplatin reduced as follows: EDTA $\geqslant$60 ml min$^{-1}$, full dose; 40–59 ml min$^{-1}$, dose of cisplatin in mg = EDTA clearance in ml min$^{-1}$; < 40 ml min$^{-1}$, no treatment.

The median number of chemotherapy cycles received by the patients was 4 (range 1–7). Treatment was continued to

Correspondence: IE Smith
Received 21 July 1994; revised 10 January 1995; accepted 24 January 1995

**Table I** Patient characteristics

| | |
|---|---|
| No. of patients | 31 |
| Sex | |
| Male | 19 |
| Female | 12 |
| Age (years) | |
| median | 53 |
| (range) | (25–68) |
| WHO PS | |
| 0 | 3 |
| 1 | 23 |
| 2 | 4 |
| 3 | 1 |
| Stage | |
| IIIB | 12 |
| IV | 19 |
| Histology | |
| Adenocarcinoma | 21 |
| Large cell | 4 |
| Squamous cell | 6 |
| No. of cycles of chemotherapy completed | |
| 1 | 3 |
| 2 | 4 |
| 3 | 6 |
| 4 | 12 |
| 5 | 0 |
| 6 | 4 |
| 7 | 2 |
| Median = 4 cycles | |

six cycles for those patients who achieved an objective response and/or symptomatic relief, but discontinued earlier in the event of progressive disease or unacceptable toxicity.

Patients developing palmar–plantar erythema on continuous infusion 5-FU were treated initially with pyridoxine 50 mg t.d.s. If this did not settle 5-FU was discontinued for 1 week and restarted with a 50 $mg\,m^{-2}$ dose reduction. If patients developed mucositis or diarrhoea, 5-FU was discontinued until symptoms subsided, and restarted with a 50 $mg\,m^{-2}$ dose reduction.

### *Assessment of response and toxicity*

The study end points were response, toxicity and survival. All patients underwent pretreatment physical examination, measurement of full blood count, plasma electrolytes, urea and creatinine, serum liver function tests, [51Cr]EDTA clearance and chest radiography. Computerised tomography was carried out when the disease was not easily evaluable on chest radiographs. Patients had chest radiography, full blood count and serum biochemistry before each treatment and were assessed at this time for objective response and toxicity according to standard WHO criteria (Miller *et al*., 1981). Complete response (CR) was defined as the disappearance of all known disease for at least 4 weeks. Partial response (PR) was defined as a reduction in the product of two diameters of the tumour by at least 50% for at least 4 weeks. Stable disease (SD) was defined as a less than 50% decrease or less than 25% increase in the size of the measurable disease without the appearance of new lesions.

Response duration and survival were calculated from the date of first treatment using the standard life table method of Kaplan and Meier (1958).

### *Assessment of symptomatic response*

Tumour-related symptoms were recorded at the start of treatment under the general headings malaise, pain, cough, dyspnoea or other, which was the specified. Symptoms were then reassessed following each course of treatment with patients asked to grade change in symptoms using simple descriptive criteria as follows: (i) complete disappearance of symptoms (CR); (ii) good improvement of symptoms (PR); (iii) minor or no change in symptoms (NC); (iv) worse (PD).

### *Ethical considerations*

This study was conducted in accordance with the Declaration of Helsinki and was approved by the Royal Marsden Hospital Ethical Committee. Witnessed, informed consent was obtained from all patients according to guidelines laid down by the committee.

### Results

### *Response and survival*

Nine of 31 patients (29%; 95% CI 13–45%) achieved an objective response with one CR and eight PRs. Six patients progressed on chemotherapy, two patients dying of progressive disease before completion of the first treatment cycle. Four of 12 patients (33%) with locally advanced disease (stage IIIB) responded compared with 5 of 17 patients (29%) with metastatic disease (stage IV). Median duration of response was 11 months. Details of response by stage are shown in Table II. Median survival for all patients was 7 months (6 months for IIIB patients, 4 months for stage IV patients).

Four out of 12 patients with stage IIIB disease responded. One of these attained a complete remission but she became severely depressed and was unable to tolerate any further local treatment. One patient attained a partial remission, down-staging her tumour sufficiently to enable lobectomy to be carried out. Unfortunately, she developed a cerebral metastasis shortly after her operation and died 3 months later. One patient responded but was not felt to be fit enough to tolerate radical radiotherapy. The other responding patient received a full course of radical radiotherapy.

### *Symptomatic response*

Twenty-one patients had complete disappearance or good improvement in at least one tumour-related symptom (70%; 95% CI 54–86%). Eight of 12 patients (67%) with stage IIIB disease had a symptomatic response, compared with 13/18 patients (72%) with stage IV disease. Response for specific symptoms was as follows: malaise, 7/15 patients (47%); pain, 11/20 patients (55%); cough, 15/21 patients (71%); dyspnoea, 13/23 patients (57%). Seven patients (23%) had progression of symptoms during chemotherapy. Details of symptomatic response are given in Table III.

Eighteen of 21 responding patients (86%) had a symptomatic response after two courses of chemotherapy. Ten of these had further symptomatic improvement with more treatment. Only three patients who failed to achieve symptomatic relief with two courses of treatment (14%) gained symptomatic response with further treatment, all achieving maximum response after three cycles.

**Table II** Tumour response by stage

| | *CR* | *PR* | *Overall response* | *SD* | *PD* | *Total No.* |
|---|---|---|---|---|---|---|
| IIIB | 1 | 3 | (33%) | 7 | 1 | 12 |
| IV | – | 5 | (26%) | 9 | 3 | 19 |
| Overall | 1 | 8 | (29%) | 16 | 4 | 31 |

**Table III** Overall symptomatic response

| *Stage* | *CR* | *PR* | *NC* | *PD* | *Total no.* |
|---|---|---|---|---|---|
| IIIB | 2 | 6 | – | 4 | 12 |
| IV | 5 | 8 | 2 | 3 | 18 |
| Total | 7(23%) | 14(47%) | 2(7%) | 7(23%) | 30 |

Seven of nine patients (78%) with an objective response to treatment gained symptomatic relief. However, 14 of 21 patients (67%) with no change or progressive disease on objective response assessment also gained symptomatic relief.

### *Toxicity*

This regimen had a low incidence of bone marrow toxicity, with only one patient developing WHO grade III/IV neutropenia and four developing WHO grade III/IV thrombocytopenia. Two patients developed significant neutropenic infection, but there were no toxic neutropenic deaths. The most significant non-haematological toxicity was emesis, with 12 patients (41%) developing grade III/IV nausea or vomiting. Although seldom severe, diarrhoea, mucositis, and palmar–plantar erythema were experienced by 31%, 34% and 14% of patients respectively. Only two patients developed significant alopecia, and there were few other significant side-effects. A detailed outline of the toxicity profile is given in Table IV.

### *Dose reductions*

Eight patients (26%) required a 25% dose reduction of at least one drug. In two cases cisplatin dose was reduced due to a reduction in EDTA clearance below 60 ml $min^{-1}$. In three patients 5-FU was reduced because of palmar–plantar erythema, and in three patients because of grade III thrombocytopenia. No patient required cessation of treatment because of treatment-induced toxicity.

### *Hickman line complications*

Seven patients (23%) experienced significant Hickman line complications, in six of whom the line required removal. Two patients developed grade III line infections and three patients developed subclavian venous thrombosis requiring line removal, one of whom also developed a pulmonary embolus. In two patients the catheter slipped from its original position into the jugular vein and needed to be removed. One of these patients also developed a pneumothorax post-Hickman line insertion, requiring insertion of a chest drain.

### Discussion

The use of 5-FU as a single agent in NSCLC has shown little activity with response rates of less than 10% (Selawry, 1973; Kris *et al.*, 1985) and there has seemed little rationale for its use in this disease when given as a bolus injection. There have been two previous reports of the use of 5-fluorouracil as a short infusion in combination with cisplatin and etoposide in advanced NSCLC, neither suggesting an advantage in terms of either response rate or survival compared with previous established cisplatin-based regimens (Flaherty *et al.*, 1991; Lynch *et al.*, 1993). Continuous infusional 5-FU, however, has not previously been assessed as front-line therapy in this setting. Giving 5-FU as a continuous infusion allows increased dose intensity without a significant increase in toxicity, and recent results in colon cancer (Lokich *et al.*, 1989), gastric cancer (Findlay *et al.*, 1994) and breast cancer (Smith *et al.*, 1993) suggest increased activity with this approach. This study was designed to see if a similar approach in combination with other known active agents would lead to a regimen with improved efficacy in advanced NSCLC.

The response rate obtained with this regimen does suggest significant activity in this tumour type, however it appears no more effective than other simpler cisplatin-based regimens (Ruckdeschel *et al.*, 1986; Cullen *et al.*, 1988; Hardy *et al.*, 1989). Although haematological toxicity was minimal, there were a number of troublesome side-effects associated with MCF. These included a significant proportion of patients with moderate to severe emesis, and the development of mucositis, diarrhoea and palmar–plantar syndrome (all related to the infusional 5-FU). The incidence of serious Hickman line complications was also greater than we had anticipated, although we may have been unlucky as a similar incidence has not been seen in other reported studies using infusional treatment in other tumour types (Lokich *et al.*, 1989; Smith *et al.*, 1993; Findlay *et al.*, 1994). Nevertheless, these toxicities all impinge on patient quality of life while on chemotherapy, an important consideration in the development of a regimen primarily concerned with palliation and symptom control.

The overall symptomatic response rate in this study was 70%. This confirms previous studies at our institution suggesting that approximately two-thirds of patients achieve useful symptomatic benefit with palliative chemotherapy, including patients who may not have responded according to objective response criteria (Hardy *et al.*, 1989; Ellis *et al.*, 1995). As in these studies, the vast majority of patients attained symptomatic relief after two courses of chemotherapy, with only a small number achieving a response after further treatment. This reinforces our impression that, in general, palliative chemotherapy should be stopped after two cycles in patients who have not achieved significant symptomatic relief.

Although active in advanced non-small-cell lung cancer and offering useful symptomatic relief in a significant proportion of patients, the toxicity profile and complexity of MCF suggests little benefit over other previously reported regimens. We therefore feel that there is little to be gained by taking this regimen forward into large randomised studies.

**Table IV** Toxicity – worst grade for all courses

| | WHO grade | | | | | | |
|---|---|---|---|---|---|---|---|
| | *0* | *1* | *2* | *3* | *4* | *%1–2* | *%3–4* |
| *Haematological* | | | | | | | |
| Anaemia | 5 | 7 | 18 | 1 | – | 81 | 3 |
| Leucopenia | 18 | 5 | 7 | 1 | – | 39 | 3 |
| Thrombocytopenia | 19 | 1 | 7 | 1 | 3 | 26 | 13 |
| *Non-haematological* | | | | | | | |
| Emesis | 2 | 7 | 8 | 10 | 2 | 52 | 41 |
| Infection | 19 | 4 | 4 | – | 2 | 28 | 7 |
| Mucositis | 19 | 7 | 2 | 1 | – | 31 | 3 |
| Diarrhoea | 20 | 7 | 2 | – | – | 31 | – |
| Alopecia | 15 | 5 | 7 | 2 | – | 41 | 7 |
| Neuropathy | 28 | 1 | – | – | – | 3 | – |
| Nephropathy | 22 | 6 | – | 1 | – | 21 | 3 |
| Palmar–plantar | 25 | 2 | 2 | – | – | 14 | – |

**Acknowledgements**

We wish to thank our secretaries Julia Holborn and Alison Norton for their help in the preparation of this manuscript. We also acknowledge the close clinical collaboration of our consultant colleagues in chest medicine and surgery, including in particular Dr Andrew Miller and Dr Rupert Courtenay-Evans (Mayday Hospital, Croydon); Dr Geoff Knowles (Kingston Hospital); Dr Nigel Cooke and Dr Paul Jones (St Helier Hospital, Carshalton); Dr Peter Mitchell-Heggs (Epsom District Hospital; Dr Paul Jenkins (East Surrey Hospital, Redhill) and Mr Norman Wright (St George's Hospital, Tooting).

### References

CARTEI G, CARTEI F, CANTONE A, CAUSARANO D, GENCO G, TOBALDIN A, INTERLAND G AND GIRALDI T. (1993). Cisplatin–cyclophosphamide–mitomycin combination chemotherapy with supportive care versus supportive care alone for the treatment of metastatic non-small cell lung cancer. *J. Natl Cancer Inst.*, **85**, 794–800.

CORMIER Y, BERGERON D, LA FORGE J, LAVANDIER M, FOURNIER M, CHENARD J AND DESMEULES M. (1982). Benefits of polychemotherapy in advanced non small cell lung bronchogenic carcinoma. *Cancer*, **50**, 845–849.

CULLEN MH, JOSHI R, CHETIYAWARDANA AD AND WOODROFFE CM. (1988). Mitomycin, ifosfamide and cisplatin in non-small cell lung cancer: treatment good enough to compare. *Br. J. Cancer*, **58**, 359–361.

ELLIS PA, SMITH IE, HARDY JR, NICOLSON MC, TALBOT DC, ASHLEY SE AND PRIEST K. (1995). Symptom relief with MVP (mitomycin C, vinblastine and cisplatin) chemotherapy in advanced non-small cell lung cancer. *Br. J. Cancer*, **71**, 366–370.

FINDLAY M, CUNNINGHAM D, NORMAN A, MANSI J, NICOLSON M, HICKISH T, NICOLSON V, NASH A, SACKS N, FORD H, CARTER R AND HILL A. (1994). A phase II study in advanced gastro-esophageal cancer using epirubicin and cisplatin in combination with continuous infusion 5-fluorouracil (ECF). *Ann. Oncol.*, **5**, 609–616.

FLAHERTY L, WOZNIAK A, REDMAN B, KRAUT M, MARTINO S, HEILBRUN L AND VALDIVIESO M. (1991). 5-Fluorouracil, etoposide and cisplatin in the management of metastatic non-small cell lung cancer. *Cancer*, **68**, 944–947.

HARDY JR, NOBLE T AND SMITH IE. (1989). Symptom relief with moderate dose chemotherapy (mitomycin C, vinblastine and cisplatin) in advanced non-small-cell lung cancer. *Br. J. Cancer*, **60**, 764–766.

KAPLAN EL AND MEIER P. (1958). Non parametric estimation from incomplete observation. *J. Am. Stat. Assoc.*, **53**, 457–481.

KRIS M, COHEN E AND GRALLA R. (1985). An analysis of 134 phase II trials in non-small cell lung cancer. (Abstract). Proceedings of the IVth World Conference on Lung Cancer, Toronto.

LOKICH JJ, AHLGREN JD, GULLO J, PHILLIPS J AND FRYER J. (1989). A prospective randomised comparison of continuous infusion fluorouracil with a conventional bolus schedule in metastatic colorectal carcinoma. A Mid Atlantic Oncology Program Study. *J. Clin. Oncol.*, **7**, 425–432.

LUEDKE DW, EINHORN L, OMURA GA, SORMA PR, BARTOLUCCI AA, BIRCH R AND GRECO FA. (1990). A randomised comparison of two combination regimens versus minimal chemotherapy in non small cell lung cancer. A South Eastern Cancer Study Group Trial. *J. Clin. Oncol.*, **8**, 886–891.

LYNCH TJ, KASS F, KALISH LA, ELIAS AD, STAUSS G, SHULMAN LN, SUGARBAKER DJ, SKARIN A AND FREI E. (1993). Cisplatin, 5-fluorouracil and etoposide for advanced non-small cell lung cancer. *Cancer*, **71**, 2953–2957.

MILLER AB, HOOGSTRATEN B, STAQUET M AND WINKLER A. (1981). Reporting results of cancer treatment. *Cancer*, **47**, 207–214.

MOUNTAIN CF. (1986). A new international staging system for lung cancer. *Chest*, **89**, 225–232.

RAPP E, PATER JL, WILLAN A, CORMIER Y, MURRAY N, EVANS WK, HODSON DI, CLARK DA, FELD R, ARNOLD AM, AYOUB JI, WILSON KS, LATREILLE J, WIERZBICKI R AND HILL DD. (1988). Chemotherapy can prolong survival in patients with advanced non small cell lung cancer – Report of a Canadian multicentre randomised trial. *J. Clin. Oncol.*, **6**, 633–641.

RUCKDESCHEL JC, FINKLESTEIN DM, ETTINGER DS, CREECH RH, MASON BA, JOSS RA AND VOGL S. (1986). A randomised trial of the four most active regimens for metastatic non small cell lung cancer. *J. Clin. Oncol.*, **4**, 14–22.

SELAWRY OS. (1973). Monochemotherapy of bronchogenic carcinoma with special reference to cell type. *Cancer Chemother. Rep.* (Part 3), **4**, 177–188.

SMITH IE, JONES AL, O'BRIEN MER, McKINNA JA, SACKS N AND BAUM M. (1993). Primary (neo-adjuvant) chemotherapy for operable breast cancer. *Eur. J. Cancer*, **29A**, 1796–1799.

SOUQUET PJ, CHAUVIN F, BOISSEL JP, CELLERINO R, CORMIER Y, GANZ PA, KAASA S, PATER JL, QUOIX E, RAPP E, TUMARELLO D, WILLIAMS J, WOODS BL AND BERNARD JP. (1993). Polychemotherapy in advanced non-small cell lung cancer: a meta-analysis. *Lancet*, **342**, 19–21.

VERONESI A, MAGRI MD AND TIRELLI U. (1988). Chemotherapy of advanced non-small cell lung cancer with cyclophosphamide, adriamycin, methotrexate and procarbazine versus cisplatin and etoposide: a randomised study. *Am. J. Clin. Oncol.*, **11**, 566–571.